# Microbiome of *Penaeus vannamei* Larvae and Potential Biomarkers Associated With High and Low Survival in Shrimp Hatchery Tanks Affected by Acute Hepatopancreatic Necrosis Disease

**DOI:** 10.3389/fmicb.2022.838640

**Published:** 2022-05-09

**Authors:** Guillermo Reyes, Irma Betancourt, Betsy Andrade, Fanny Panchana, Rubén Román, Lita Sorroza, Luis E. Trujillo, Bonny Bayot

**Affiliations:** ^1^Centro Nacional de Acuicultura e Investigaciones Marinas (CENAIM), Escuela Superior Politécnica del Litoral (ESPOL), Guayaquil, Ecuador; ^2^Facultad de Ciencias de la Vida (FCV), Escuela Superior Politécnica del Litoral (ESPOL), Guayaquil, Ecuador; ^3^Facultad de Ciencias Agropecuarias, Universidad Técnica de Machala, Machala, Ecuador; ^4^Industrial Biotechnology Research Group, Center for Nanoscience and Nanotechnology (CENCINAT), Universidad de las Fuerzas Armadas (ESPE), Sangolquí, Ecuador; ^5^Facultad de Ingeniería Marítima y Ciencias del Mar (FIMCM), Escuela Superior Politécnica del Litoral (ESPOL), Guayaquil, Ecuador

**Keywords:** AHPND, biomarkers, differential abundance, microbiome, *Penaeus vannamei* larvae, 16S rRNA gene

## Abstract

Acute hepatopancreatic necrosis disease (AHPND) is an emerging bacterial disease of cultured shrimp caused mainly by *Vibrio parahaemolyticus*, which harbors the lethal PirAB toxin genes. Although *Penaeus vannamei* (*P. vannamei*) postlarvae are susceptible to AHPND, the changes in the bacterial communities through the larval stages affected by the disease are unknown. We characterized, through high-throughput sequencing, the microbiome of *P. vannamei* larvae infected with AHPND-causing bacteria through the larval stages and compared the microbiome of larvae collected from high- and low-survival tanks. A total of 64 tanks from a commercial hatchery were sampled at mysis 3, postlarvae 4, postlarvae 7, and postlarvae 10 stages. PirAB toxin genes were detected by PCR and confirmed by histopathology analysis in 58 tanks. Seven from the 58 AHPND-positive tanks exhibited a survival rate higher than 60% at harvest, despite the AHPND affectation, being selected for further analysis, whereas 51 tanks exhibited survival rates lower than 60%. A random sample of 7 out of these 51 AHPND-positive tanks was also selected. Samples collected from the selected tanks were processed for the microbiome analysis. The V3–V4 hypervariable regions of the 16S ribosomal RNA (rRNA) gene of the samples collected from both the groups were sequenced. The Shannon diversity index was significantly lower at the low-survival tanks. The microbiomes were significantly different between high- and low-survival tanks at M3, PL4, PL7, but not at PL10. Differential abundance analysis determined that biomarkers associated with high and low survival in shrimp hatchery tanks affected with AHPND. The genera *Bacillus*, *Vibrio*, *Yangia*, *Roseobacter*, *Tenacibaculum*, *Bdellovibrio*, *Mameliella*, and *Cognatishimia*, among others, were enriched in the high-survival tanks. On the other hand, *Gilvibacter*, *Marinibacterium*, *Spongiimonas*, *Catenococcus*, and *Sneathiella*, among others, were enriched in the low-survival tanks. The results can be used to develop applications to prevent losses in shrimp hatchery tanks affected by AHPND.

## Introduction

Diseases that affect the early larval stages of farmed shrimp are one of the main limitations for the sustainability of the shrimp industry. Thus, it is essential to implement mitigation tools, such as administering probiotics for bacterial infections. Some genera and species such as *Vibrio harveyi* (*V. harveyi*) (Haldar et al., 2011; Mirbakhsh et al., 2014), *Vibrio alginolyticus* (*V. alginolyticus*) (Karunasagar et al., 1998; Hasan et al., 2017), and *Vibrio campbellii* (*V. campbellii*) (Hameed, 1995; Soto-Rodríguez et al., 2006; Haldar et al., 2011) have been reported as recurrent pathogens in commercial shrimp hatcheries from many producing countries. Acute hepatopancreatic necrosis disease (AHPND), caused mainly by *Vibrio parahaemolyticus* (Tran et al., 2013), produces massive mortalities (Kongrueng et al., 2015; Sirikharin et al., 2015) and affects cultured *Penaeus vannamei* (*P. vannamei*) and *Penaeus monodon* (*P. monodon*) shrimps (de la Peña et al., 2015). AHPND-causing strains contain plasmids of approximately 70 kbp and harbor genes encoding for two toxins related to the PirA and PirB insecticidal toxin (Han et al., 2015). The PirAB toxins are released in the digestive system of the shrimp, causing severe desquamation of the hepatopancreas cells and the consequent death of the infected shrimp.

The diversity of species conforming to the intestinal microbiome performs critical functions in a host, such as aiding nutrient absorption, antagonizing against pathogenic bacteria, and improving the immune system (Dai et al., 2020). Therefore, the studies on the intestinal microbiome are helpful to identify biomarkers that could predict the onset of diseases through comparative analysis. For example, in a study of the shrimp microbiome throughout early stages, the genus *Cupriavidus* was always present and significantly abundant in healthy larvae (Zheng et al., 2017). Similarly, *Meridianimaribacter* has been identified in healthy shrimp water (Xue et al., 2015). Recently, the function and assembly of the bacterial community and biomarkers associated with each developmental stage for shrimp *P. vannamei* have been reported (Wang H. et al., 2020). Most investigations have characterized the *P. vannamei* microbiome in juvenile stages (Chen et al., 2017; Cornejo-Granados et al., 2017; Xiong et al., 2018; Dong et al., 2021; Restrepo et al., 2021). Thus, the microbiome of juvenile *P. vannamei* shrimp affected by AHPND is significantly different than in healthy shrimp (Cornejo-Granados et al., 2017). However, little is known about the bacterial community in larval stages under AHPND affectation.

This study aimed to characterize, through high-throughput sequencing (HTS), the microbiome of *P. vannamei* naturally infected with AHPND-causing bacteria across the larval stages and to obtain biomarkers associated with high and low survival in shrimp hatchery tanks affected with AHPND. The results can be used for further development of probiotics to mitigate AHPND.

## Materials and Methods

### Sample Collection and Processing

**FIGURE 1:**
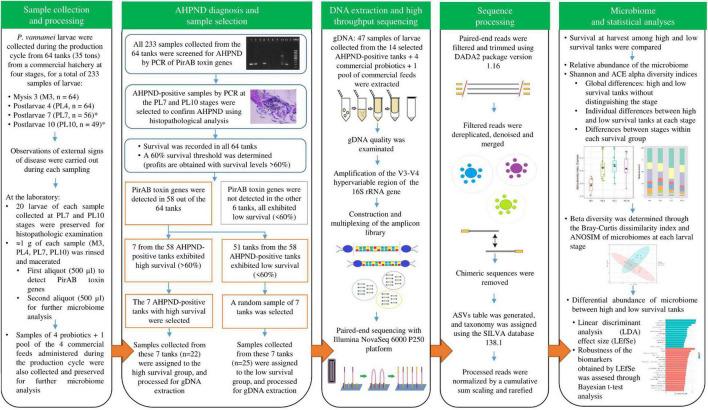
Experimental design for the microbiome characterization of *Penaeus vannamei* (*P. vannamei*) larvae affected with acute hepatopancreatic necrosis disease (AHPND) and identification of potential biomarkers associated with high and low survival in shrimp hatchery tanks through high-throughput sequencing. *Only 56 and 49 samples were collected at PL7 and PL10 as the population of 8 and 7 tanks died at PL6 and PL9 stages, respectively.

*Penaeus vannamei* larvae were collected from January to March 2021, during the whole production cycle, from 64 tanks (35 tons) from a commercial hatchery in South America (Figure 1). Sampling was performed in each tank at four stages, for a total of 233 samples of larvae: mysis 3 (M3) (*n* = 64), postlarvae 4 (PL4) (*n* = 64), postlarvae 7 (PL7) (*n* = 56), and postlarvae 10 (PL10) (*n* = 49). Only 56 and 49 samples were collected at PL7 and PL10 as the population of 8 and 7 tanks died at PL6 and PL9 stages, respectively. All the tanks received the same management protocol: feeding, water exchange, and supplementation of commercial probiotics (Supplementary Table 1). Water quality parameters during the production cycles were: water temperature = 32.5–33.0°C, dissolved oxygen concentration = 5.0 mg/L, pH = 7.9–8.1, and salinity = 35.0 ± 0.0 g/L. Observations of external signs of disease were carried out during each sampling (swimming behavior, empty digestive tract, and larval activity). After a maximum of 1 h from sampling to processing at the laboratory, 20 larvae of each sample collected at PL7 and PL10 stages were preserved in Davidson’s AFA fixative solution for histopathologic examination. Approximately, 1 g of each sample (M3, PL4, PL7, and PL10) was rinsed with a 2% NaCl sterile solution to remove impurities and external microorganisms. The last group of samples was macerated for homogenization of the bacterial load. A first aliquot (500 μl) was processed to detect subsequent PirAB toxin genes. A second aliquot (500 μl) was distributed in 2 ml cryovial tubes to submerge them for 30 min in liquid nitrogen (−196°C) and immediately stored at −80°C for further microbiome analysis. Samples of the four commercial probiotics and a pool of the four commercial feeds administered during the production cycle (Supplementary Table 1) were also collected and preserved, as mentioned above, for further microbiome analysis.

### Acute Hepatopancreatic Necrosis Disease Diagnosis and Sample Selection

**FIGURE 2:**
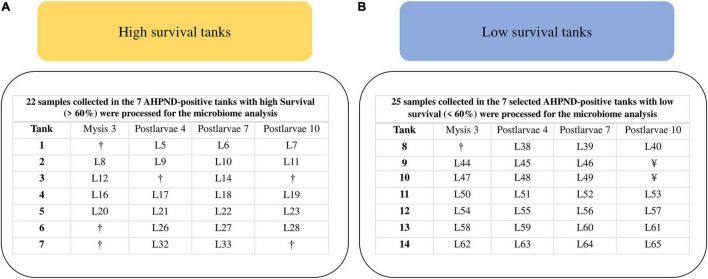
Samples selected for the microbiome characterization of *P. vannamei* larvae affected by AHPND. **(A)** High-survival tanks. **(B)** Low-survival tanks. ^†^Sample did not pass the DNA quality control. ^¥^Sample not collected as the tank population died at PL9.

All the 233 samples collected from the 64 tanks were screened for AHPND by PCR of PirAB toxin genes (Figure 1). Genomic DNA (gDNA) was extracted from each sample using the phenol standard protocol (Kerkhof and Ward, 1993). PirAB toxin genes were amplified with the primers AP4 F1/R1–AP4 F2/R2, VpPirA-284F/R, and VpPirB-392F/R (Dangtip et al., 2015; Han et al., 2015). AHPND-positive samples by PCR at the PL7 and PL10 stages were selected to confirm AHPND affectation using histopathological analysis. The paraffin-embedded tissue sections were cut at 4 μm, stained with Mayer–Bennet H&E (Bell and Lightner, 1988), and examined for histopathological changes. Survival was recorded in all the 64 tanks: at harvest in 49 tanks (PL11) and reported as 0% for the other 15 tanks, as their populations died at PL6 (8 tanks) and PL9 (7 tanks). A 60% survival threshold was determined according to the historical data of the shrimp hatchery, where profits were obtained with survival levels higher than 60% (Figure 1). PirAB toxin genes were detected in 58 out of the 64 tanks and not detected in the other 6 tanks (Figure 1). These six tanks exhibited low survival (<60%). Seven from the 58 AHPND-positive tanks exhibited high survival (>60%), whereas 51 tanks exhibited low survival (<60%) (Figure 1). The seven AHPND-positive tanks with high survival were selected for further analysis (Figure 1). As the number of AHPND-positive tanks with low survival was much higher than the high ones, a random sample of seven tanks was selected for further analysis to obtain an equal number of tanks for each survival condition (Figure 1). Samples of larvae collected from these 14 AHPND-positive tanks were assigned to the two groups of survival conditions: high (survival >60%, *n* = 22 samples of larvae) and low (survival <60%, *n* = 25 samples of larvae) and processed for gDNA extraction (Figures 1, 2).

### Deoxyribonucleic Acid Extraction and High-Throughput Sequencing

The gDNA of the 47 AHPND-positive samples of larvae from the 14 selected tanks (high and low survival), four commercial probiotics, and the pool of commercial feeds were extracted (Figure 1) using the ZymoBIOMICS DNA Microprep Isolation Kit (Zymo Research, United States), following the manufacturer’s instructions. An aliquot of 20 μl of gDNA was diluted in DNase-free ultrapure water. DNA quality (1.8–2.0 A260/280) was examined through a NanoDrop One Microvolume UV-Vis Scanning Spectral Spectrophotometer (Thermo Fisher Scientific, United States). The amplification of the V3–V4 hypervariable region (470 bp) of the 16S ribosomal RNA (rRNA) gene was performed with the 341F/806R primers (Takahashi et al., 2014). DNA was submitted to Novogene Incorporation (Beijing, China) for construction and multiplexing of the amplicon library. Paired-end sequencing was performed with Illumina NovaSeq 6000 P250 platform. The sequences were deposited in the National Center for Biotechnology Information (NCBI) Sequence Read Archive (SRA) under accession number PRJNA800805.

### Sequence Processing

Paired-end reads were processed using DADA2 package version 1.16 (Callahan et al., 2016) in the R software version 3.6.3 (Figure 1). Without adapters and primers, paired-ends were filtered and trimmed using the filterAndTrim function [sequences removed with more than two expected errors—maxEE = c(2,2) and trimming of last 10 nt of the forward reads and last 20 nt of the reverse reads—truncLen = c(240,230)]. The filtered reads were dereplicated, denoised, and merged (forward and reverse amplicon sequences) to get a merging sequence. Then, the chimeric sequences were removed using the “consensus” method from the removeBimeraDenovo function. The table of amplicon sequence variants (ASVs) was generated with the makeSequenceTable function and the taxonomy was assigned with the assignTaxonomy function using the public SILVA database version 138.1. A total number of 7 million clean reads was obtained (Supplementary Table 2), with an average of 149,047 clean reads per sample (range: 130,040–168,784). The error rate averaged 0.03% for all the reads. On average, 88% of the reads exhibited a Phred quality (*Q* > 30) and a GC content of 54%. A reading length of 250 bp was averaged for the alignment. A total of 8,626 ASVs was obtained, with an average of 393 ASVs per sample. The processed reads were normalized by a cumulative sum scaling and rarefied to 77,220 sequences per sample, which was sufficient to capture the alpha-diversity of the microbial communities through all the larval stages. Good’s coverage was higher than 99.99%, indicating an optimal sequencing depth.

### Microbiome and Statistical Analyses

The survivals at harvest among the high- and low-survival tanks were compared by the *t*-test (Figure 1). Previously, the variance homogeneity and assumption of normality of both the treatments were examined through the F and Shapiro–Wilk normality tests. The null hypothesis, equal survival at both the tank groups, was rejected with a *p* < 0.05.

The microbiome analyses were performed at the ASVs level. The variability of the alpha-diversity indices and relative abundance of the microbiome were studied for the four stages from the high- and low-survival tanks (Figure 1). The alpha-diversity of the larval microbiome was estimated through the Shannon (Shannon and Weaver, 1949) and abundance-based coverage estimator—ACE (Hughes et al., 2001) indices. Three statistical analyses were carried out to evaluate the hypotheses of each alpha-diversity index. The first statistical analysis evaluated the global differences between the high- and low-survival tanks without distinguishing the stage. The second analysis evaluated individual differences between the high- and low-survival tanks at each stage. Both the analyses were performed with the *t*-tests. Previously, the variance homogeneity and assumption of normality of treatments were examined through the F and Shapiro–Wilk tests. The third statistical analysis evaluated differences between stages within each survival condition group using the one-way ANOVA. The variance homogeneity and assumption of normality of treatments were previously examined through the Bartlett and Shapiro–Wilk tests. When significant results were found in the ANOVA test, the pairwise Tukey’s honestly significant difference test for multiple comparisons was used to evaluate statistical differences of the correspondent alpha-diversity index between stages. The null hypothesis, equal alpha-diversity indices between the groups, was rejected with a *p* < 0.05.

The beta-diversity was determined through the multivariate analysis of the Bray–Curtis dissimilarity index and visualized through a principal coordinate analysis (PCoA) for each stage (Figure 1). The null hypothesis of equal microbiomes between the high- and low-survival tanks was evaluated by the analysis of similarities (ANOSIM) at each larval stage.

Differential abundance analysis of the larval microbiome between the high- and low-survival tanks (Figure 1) was performed using a linear discriminant analysis (LDA) effect size (LEfSe) (Segata et al., 2011). A *p*-value correction for the multiple hypotheses, simultaneously tested at the LEfSe analysis, was performed to control the false discovery rate (FDR) (proportion of type I error) and increase the power to detect true positive biomarkers (Nearing et al., 2021; Wallen, 2021). ASVs with LDA cutoff of 2.0 and significant differences (*p* < 0.05) were considered as potential biomarkers associated with high and low survival in shrimp hatchery tanks affected with AHPND. To assess the robustness of the biomarkers obtained with the LEfSe analysis, a Bayesian *t*-test analysis was also performed for each biomarker associated either to the high- or low-survival tanks. The predictive performances of the null hypothesis H_0_ (equal ASVs abundance between the high- and low-survival tanks) and alternative hypothesis H_1_ (different ASVs abundance between the high- and low-survival tanks) were compared. The hypothesis testing was performed through the Bayes factor BF_10_, where BF_10_ indicates the relative performance in favor of H_1_ over H_0_ provided by the data and calculated as the ratio of the probability of H_1_ over the probability of H_0_ based on the data. Bayes factors higher than 1 showed evidence in favor of H_1_, with ranges of 1–3, 3–10, and >10 interpreted as weak, moderate, and strong evidence in favor of H_1_ (van Doorn et al., 2021). Microbiome and statistical analyses were conducted with the MicrobiomeAnalyst (Dhariwal et al., 2017), microbiomeMarker (Cao, 2020), Stats (R Core Team, 2013), and BayesFactor (Morey and Rouder, 2011) packages implemented in the R software version 3.6.3.

## Results

### Acute Hepatopancreatic Necrosis Disease Diagnosis and Sample Selection

PirAB toxin genes were detected in 58 out of 64 sampled tanks, whereas 6 tanks were negative. Larvae from the positive tanks presented symptoms of diseases (erratic swimming and pale hepatopancreas), especially at PL4 and PL7. The histopathologic analysis of these larvae samples at PL7 and PL10 revealed AHPND lesions at the hepatopancreas. Seven tanks from the 58 AHPND-positive tanks exhibited survival rates higher than 60% at harvest (mean ± SEM = 67.8 ± 3.10%), despite the AHPND affectation. The 22 samples of larvae collected from these 7 AHPND-positive tanks were assigned to the group of high-survival tanks and processed for gDNA extraction (Figure 2). The other 51 AHPND-positive tanks presented survival rates (15.2 ± 1.7%) lower than 60%. The random sample of 7 tanks selected from these 51 tanks exhibited an average survival of 17.9 ± 4.6% at harvest. The 25 samples of larvae collected from these 7 AHPND-positive tanks were assigned to the group of low-survival tanks and processed for gDNA extraction (Figure 2). A significant (*p* < 0.001) survival difference of 49.9% was observed between the high- and the selected low-survival tanks at harvest. The prevalences of AHPND lesions (mean ± SEM) in the larvae were 40.4 ± 13.6% in the high-survival tanks and 61.3 ± 13.2% in the low-survival tanks.

### Microbiome and Statistical Analyses

**FIGURE 3:**
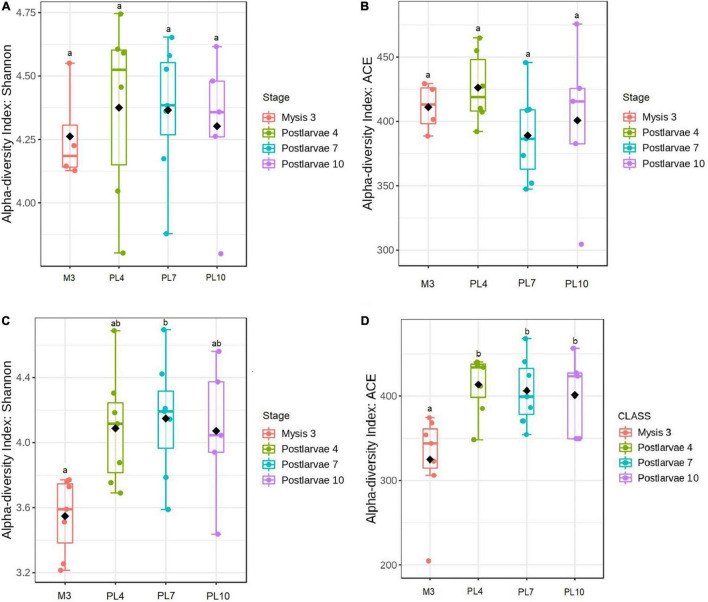
Alpha-diversity of the larval microbiome at amplicon sequence variant (ASV) level in the high- and low-survival tanks affected by AHPND. **(A)** Shannon index for the high-survival tanks. **(B)** ACE index for the high-survival tanks. **(C)** Shannon index for the low-survival tanks. **(D)** ACE index for the low-survival tanks. At each survival condition, pair of larval stages with different letters indicates alpha-diversity indices significantly different at *p* < 0.05, based on the Tukey’s honestly significant difference test.

In the first statistical analysis, significant differences (*p* = 0.004) in the Shannon index were found between the high- and low-survival tanks without distinguishing the stage (Supplementary Figure 1A); however, the ACE index was non-significantly different (*p* = 0.273) between both the groups (Supplementary Figure 1B). In the second statistical analysis, significantly lower Shannon and ACE indices (*p* = 0.002 and *p* = 0.030, respectively) were observed at M3 between the high- and low-survival tanks (Supplementary Figures 2A,B). Non-significant differences (*p* > 0.055) in the Shannon and ACE diversity indices were found when the samples from the high- and low-survival tanks were compared at the other three stages (Supplementary Figures 2A,B). In the third analysis, non-significant differences (*p* = 0.933 and *p* = 0.538, respectively) of the Shannon and ACE diversity indices were identified within the high-survival tanks (Figures 3A,B). However, the Shannon index was significantly lower (*p* = 0.038) at M3 than PL7 at the low-survival tanks (Figure 3C). The ACE index was significantly lower (*p* < 0.026) at M3 compared with the other three stages (Figure 3D), but non-significantly different (*p* > 0.998) between the other three stages at low-survival tanks (Figure 3D).

Eighteen phyla, 29 classes, 74 orders, 107 families, 180 genera, and 58 species were identified in all the larva samples.

**FIGURE 4:**
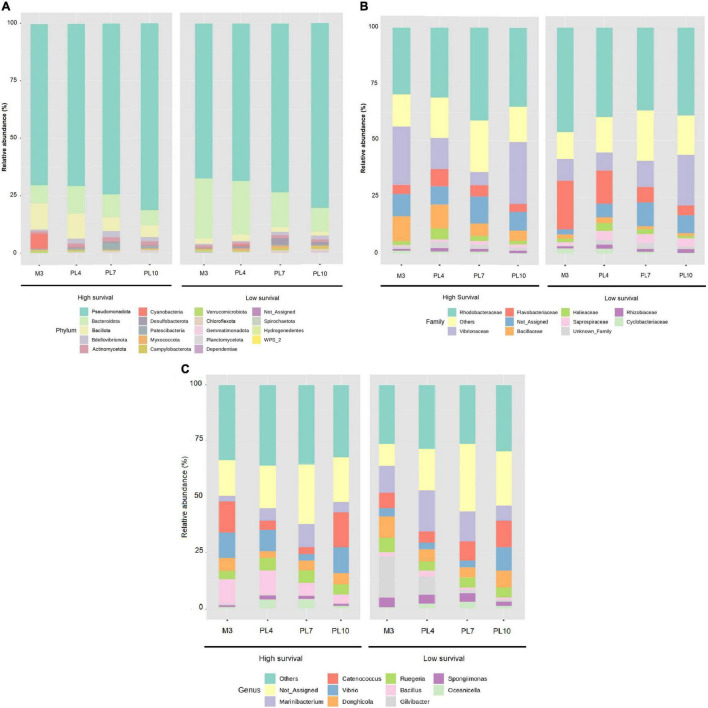
Relative abundance of the larval microbiome in the high- and low-survival tanks affected with AHPND. **(A)** Phylum level. **(B)** Family level. **(C)** Genus level.

At the phylum level, *Pseudomonadota* exhibited the highest, and similar, abundances at both the high (74.2%) and low (73.0%) survival tanks, with 73.6% across the four stages at both the survival conditions (Figure 4A and Supplementary Table 3). *Bacteroidota* and *Bacillota* followed global average abundances of 13.6 and 5.2%, respectively (Figure 4A and Supplementary Table 3).

The *Rhodobacteraceae* family exhibited the highest dominance compared to other families at both the high- and low-survival tanks (Figure 4B and Supplementary Table 3). The *Vibrionaceae* was the second most abundant family, especially at PL10 (41%), and at M3 (25%), in the high-survival tanks, contrasted with the same stages at the low-survival tanks (23 and 11%, respectively, Figure 4B and Supplementary Table 3). In contrast, *Flavobacteriaceae* was more abundant, especially at M3 and PL4, in the low-survival tanks, six and twofold higher than the abundances reported at the same stages in the high-survival tanks (Figure 4B and Supplementary Table 3). *Bacillaceae* showed the highest dominance in the high-survival tanks, five and twofold higher at M3 and PL4 than the low-survival tanks (Figure 4B and Supplementary Table 3).

The genus *Vibrio* was observed to be increased at M3 and PL4 stages in the high-survival tanks (Figure 4C and Supplementary Table 3), while *Bacillus* was more abundant in the high compared with the low-survival tanks (Figure 4C and Supplementary Table 3). The abundance of the *Marinibacterium* and *Gilvibacter* genus across stages was higher in the low-survival tanks compared with the high one (Figure 4C and Supplementary Table 3).

The dominant phyla in the probiotics samples were *Bacillota* (87%) and *Pseudomonadota* (11%) (Supplementary Figure 3A). At the genus level, the commercial probiotics 1, 2, and 3 samples were dominated by the genus *Lentilactobacillus*, *Bacillus*, and *Clostridium* (Supplementary Figure 3B). The probiotic four sample was dominated by the genus *Weissella* and *Bacillus* (Supplementary Figure 3B).

The phyla *Bacillota* (58%), *Pseudomonadota* (33%), and *Cyanobacteria* (10%) dominated the commercial feed sample (Supplementary Figure 3A). At the genus level, *Lactobacillus* and *Bacillus* were the most dominant in the commercial feed sample (Supplementary Figure 3B).

**FIGURE 5:**
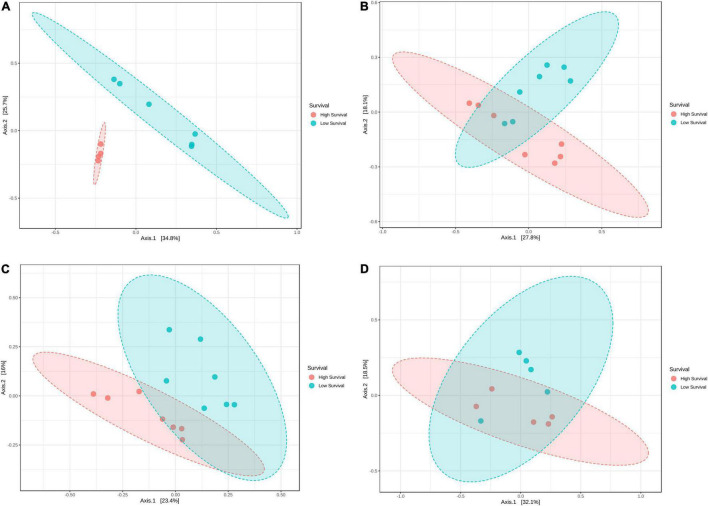
Principal coordinate analysis (PCoA) of the Bray–Curtis dissimilarity index from larvae collected at the high- and low-survival tanks affected with AHPND. **(A)** Mysis 3. **(B)** Postlarvae 4. **(C)** Postlarvae 7. **(D)** Postlarvae 10.

The ANOSIM analysis showed that the microbiomes were significantly different (*p* = 0.001) between the high- and low-survival tanks, with significant differences (*p* < 0.012) at M3, PL4, and PL7 (Figure 5). Such difference was more evident at M3 (Figure 5). Non-significant differences (*p* = 0.112) were found in the microbiomes at PL10 comparing both the survival conditions (Figure 5).

**FIGURE 6:**
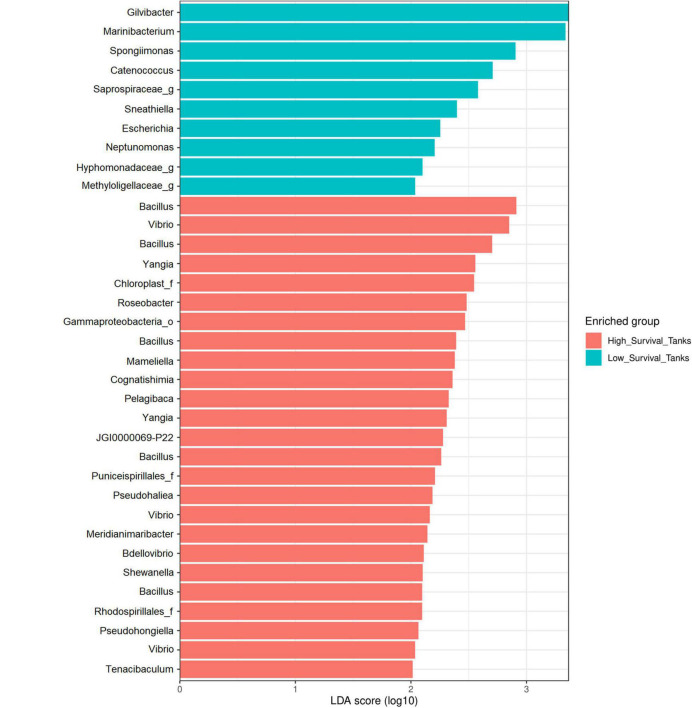
Results of the differential abundance analysis using a linear discriminant analysis (LDA) effect size (LEfSe) of larvae collected from the high- and low-survival tanks affected with AHPND. The length of the bar represents the effect size (LDA cutoff = 2) of all the bacterial lineages at the genus level.

**FIGURE 7:**
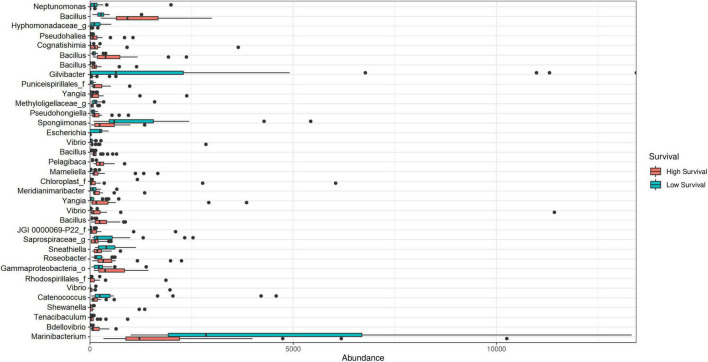
Absolute abundance of ASV biomarkers at the genus level at the high- and low-survival tanks affected with AHPND.

The LEfSe analysis showed 35 significant ASVs, 25 of which were differentially abundant for the high-survival tanks and 10 for the low-survival tanks (Supplementary Table 4 and Supplementary Figure 4). Consistently, the Bayesian analysis showed evidences (strong and moderate) for difference in the ASVs abundances between the high- and low-survival tanks for 33 ASVs (Supplementary Table 5). Only for two AVSs (ASV_58 and ASV_152), a weak evidence in favor of differences in the ASVS abundance between both the groups was reported (Supplementary Table 5). The genera *Bacillus*, *Vibrio*, *Yangia*, *Roseobacter*, *Tenacibaculum*, *Bdellovibrio*, *Mameliella*, *Cognatishimia*, and *Pelagibaca*, among others, were significantly enriched at the high-survival tanks, with *Bacillus* being the most abundant (Figures 6, 7). On the other hand, the genera *Gilvibacter*, *Marinibacterium*, *Spongiimonas*, *Catenococcus*, *Sneathiella*, and *Escherichia*, among others, were significantly enriched at the low-survival tanks (Figures 6, 7). *Gilvibacter* and *Marinibacterium* showed the largest effect size (LDA >2.0) at the low-survival tanks (Figure 6). Four ASVs differentially abundant at the high-survival tanks were also found in the probiotic samples (ASV-8, ASV-9, ASV-29, and ASV-52). These four ASVs were taxonomically identified as *Bacillus* (Figure 6 and Supplementary Figure 4). One ASV (ASV-73), differentially abundant at the high-survival tanks and identified as *Bacillus*, was not present in the probiotic samples (Figure 6 and Supplementary Figure 4). In addition, only one commercial probiotic sample (probiotic 1) did not report ASVs with significant abundance at the high-survival tanks.

## Discussion

Most studies on the *P. vannamei* microbiome have characterized the bacterial communities by comparing shrimp organs, diets, larval stages, culture systems, culture water and sediments, ecosystems, weight ranges, and stress conditions (Tzuc et al., 2014; Zhang M. et al., 2014; Zeng et al., 2017; Gainza et al., 2018; Huang et al., 2018; Zoqratt et al., 2018; Fan et al., 2019; Liu et al., 2019). Little study has been done on the shrimp microbiome under a sanitary approach, focusing primarily on juvenile stages (Chen et al., 2017; Cornejo-Granados et al., 2017). This study is part of a larger project that intends to discover effective probiotics for the control of AHPND in shrimp hatcheries. In this context, the main objectives of this study were to characterize the microbiome of *P. vannamei* larvae affected by AHPND in a commercial hatchery and to study the effect of the survival condition (high vs. low) on the microbiomes of larvae affected with AHPND to find biomarkers with the potential of being probiotics for a future AHPND control. For this reason, we did not use the control group (non-infected samples of larvae), as it would not indicate whether their microbiome would provide protection specifically for AHPND infection. The prevalence of AHPND was high and most tanks showed low survival at harvest. However, although a few AHPND-positive tanks with high survival at harvest were reported, a significant survival difference between the high- and low-survival tanks allowed the comparison of the microbiome between both the groups of samples.

We performed a description of the microbiome variability at four stages and compared two survival conditions. The Shannon diversity index was significantly lower at the low-survival tanks. ACE index at the M3 stage was significantly lower than the other stages at the low-survival tanks and lower than the same stage at the high-survival tanks. On the contrary, alpha-diversity indices were not different among stages at the high-survival tanks. Similar findings have been found by Xiong et al. (2018) and Wang Y. et al. (2020), who concluded that the success of a larval culture depends on the high bacterial diversity from the beginning of the host’s life. Also, the comparative analysis of the beta-diversity showed a significant difference in the microbiomes between both the survival conditions at M3, PL4, and PL7, but not at PL10. A plausible explanation for the difference in the microbiome structures at M3, PL4, and PL7 could be that the outbreak events occurred at the earlier stages, as larvae with symptoms of diseases were observed especially at PL4 and PL7. Samples collected at PL10, when the mortality stopped, could have been the survivors with a similar microbiome structure. Three top genera (*Vibrio*, *Marinibacterium*, and *Gilvibacter*) presented similar relative abundance in the high- and low-survival tanks at the end of the production cycle (PL10). However, *Bacillus* was less predominant in the low-survival tanks at PL10. In fact, *Bacillus* decreased over the time in the high-survival tanks. Another explanation could be that a different bacterial diversity is adhered to the dermis in the early larval stages, but through larval development, the digestive system is forming, the larvae are acquiring the necessary communities to their benefit and much of the dermal microbiome is lost, thus acquiring a more stable gut microbiome (Angthong et al., 2020; Wang H. et al., 2020; Wang Y. et al., 2020).

*Pseudomonadota* is the most abundant phylum reported for *P. vannamei* larvae and cultured water (Wang H. et al., 2020; Xue et al., 2020) for healthy and diseased larvae (Zheng et al., 2016). *Pseudomonadota* is also reported as abundant in *P. vannamei* juvenile shrimp (Xiong et al., 2015; Zeng et al., 2017) and wild and domesticated adult *P. monodon* (Rungrassamee et al., 2014). *Bacteroidota* is the second most abundant phylum reported for healthy larvae (Zheng et al., 2016) and juvenile *P. vannamei* (Wang et al., 2019) and water samples from healthy cultures (Xue et al., 2020). *Bacillota* is also reported but with a lower abundance in the *P. vannamei* larvae microbiome (Wang Y. et al., 2020) and healthy juvenile shrimp (Zeng et al., 2017; Wang et al., 2019; Restrepo et al., 2021). Our results are consistent with these observations of the highest dominance of *Pseudomonadota* at both the survival conditions, followed by a lower abundance of *Bacteroidota* and *Bacillota*. The *Bacillota*/*Bacteroidota* ratios of relative abundance were different between both the groups, with more than sevenfold higher ratio in the high-survival tanks (0.92) compared to the low ones (0.13) (Supplementary Table 3). The *Bacillota*/*Bacteroidota* ratio is an important index for several processes where the microbiota plays an important role in their host. Lower *Bacillota*/*Bacteroidota* ratios have been reported in the gut microbiome of *P. vannamei* with a low growth rate (0.06) compared to a normal (0.93) growth rate (Fan and Li, 2019). The latter ratio, which was similar to the value reported in this study for the high-survival tanks, has been explained by a better nutrient uptake in gut shrimp (Fan and Li, 2019). *Bacillota* could contribute to the energy storage of food by playing a role in the breakdown of polysaccharides. They have been reported in human and shrimp microbiota (Ley et al., 2006; Turnbaugh et al., 2006; Su et al., 2021). *Bacillota* and *Bacteroidota* also might contribute to the metabolism regulation to conserve energy during temperature changes, as higher ratios increase during temperature fluctuation. *Bacillota* is also one of the important bacterial groups for health and immunity in crustaceans (Duan et al., 2018; Foysal et al., 2019). Interestingly, we observed that the ratio *Bacillota*/*Bacteroidota* at the M3 stage was higher and exhibited more differences between the high (1.52) and low (0.09) survival tanks, which meant increase of 17-fold of the ratio *Bacillota*/*Bacteroidota* at the M3 stage at the high-survival tanks than the same stage at the low-survival tanks. Considering such a consistent pattern of decrease of the *Bacillota*/*Bacteroidota* ratio in the low-survival tanks, which was more intense at the M3 stage, it will be important to perform additional investigations to clarify the relation of both the taxa on shrimp health.

*Rhodobacteraceae* family is dominant and persistent in the digestive tract of juvenile and adult shrimp and adults. Therefore, it is considered vital in the search for probiotics (Xiong et al., 2018). We observed that *Rhodobacteraceae* was the dominant and ubiquitous family at all the larval stages. Specifically, the genera *Yangia*, *Mameliella*, *Roseobacter*, and *Pelagibaca*, which belong to this family, were significantly most enriched at the high-survival tanks. *Mameliella* is a genus involved in toxin degradation and production of metabolites, such as poly-β-hydroxybutyrate (PHB) that has beneficial effects on the immune response of *P. vannamei* (Duan et al., 2019, 2020). *Roseobacter* has been described as a suppressor of the growth of pathogenic vibrios in fish and its antibacterial effect against pathogens is enhanced by the presence of phytoplankton (Hjelm et al., 2004; Sharifah and Eguchi, 2011).

Some *Vibrio* species, such as *V. harveyi*, *V. alginolyticus*, and *V. campbellii*, are pathogenic for shrimp larvae (Karunasagar et al., 1994, 1998; Hameed, 1995; Robertson et al., 1998; Vandenberghe et al., 1998, 1999; Soto-Rodríguez et al., 2006; Haldar et al., 2011; Mirbakhsh et al., 2014; Hasan et al., 2017). Unexpectedly, the *Vibrionaceae* family was less abundant in larvae collected from the low-survival tanks. These results coincide with observations in diseased larvae (Zhang D. et al., 2014; Zheng et al., 2017) and AHPND-affected juvenile shrimp (Cornejo-Granados et al., 2017). *Vibrio* is the most important genus of the *Vibrionaceae* family in shrimp culture. It is ubiquitous in the marine environmental microbiome and, therefore, part of the shrimp culture. Moreover, *Vibrio* can be effective as a shrimp probiotic (Restrepo et al., 2021). *Vibrio* is abundant in nauplius and it is not considered a disease biomarker (Wang H. et al., 2020; Wang Y. et al., 2020). Consistently, three ASVs were enriched in the high-survival tanks (ASV-35, ASV-58, and ASV-164) and none in the low-survival tanks. In this study, we did not perform a reverse transcriptase quantitative PCR (RT-qPCR) analysis and, therefore, we could not quantify the expression of PirAB toxin genes. However, given the relevance of the AHPND control, it will important to investigate the relationship between expression of AHPND-causing bacteria and abundance and structure of other members of the *Vibrionaceae* family in the high- and low-survival tanks.

The *Flavobacteriaceae* family has been reported to be dominant in the microbiome of shrimp in metamorphosis (pre- and postlarvae) stages (Yan et al., 2020) and prevalent in culture water from zoea to early postlarvae stages (Xue et al., 2020). In this study, *Flavobacteriaceae* were more abundant at the earlier stages of M3 and PL4, especially at the low-survival tanks. After that, the abundances decreased reaching PL10. We found genera from this family at both the survival tanks meaning that the biomarker condition depends on the bacteria genus.

*Bdellovibrio* has been described to improve the growth and health of *Scophthalmus maximus* fish and *P. monodon* shrimp (Li et al., 2014; Cao et al., 2015). Also, it has the potential to reduce the biofilm formation of *Vibrio* (Guo et al., 2016), depredate pathogenic *Vibrio* of *P. vannamei* (Cao et al., 2020), and has been proposed as probiotics for *P. vannamei* shrimp (Kongrueng et al., 2017). Furthermore, in this study, an ASV assigned to the *Bdellovibrio* genus (ASV-112) was significantly more enriched in the high-survival tanks. Therefore, it is a potential candidate for shrimp probiotics, being necessary to resolve the taxonomic identification to a species level.

The *Bacillus* genus is widely described as an effective probiotic (Balcázar and Rojas-Luna, 2007; Tseng et al., 2009). Some *Bacillus* strains have significant inhibitory effects on pathogens and improve the resistance of *P. vannamei* shrimp in the presence of AHPND-causing bacteria (Kewcharoen and Srisapoome, 2019). Four ASVs found in the probiotic samples, taxonomically assigned as *Bacillus*, were also significantly enriched in the larva microbiota from the high-survival tanks. These ASVs were reported in three probiotic samples. The fourth probiotic did not show any ASVs significantly enriched in the high-survival tanks. The fact that the four *Bacillus* biomarkers from the probiotics were only detected in the high-survival tanks, despite the administration of probiotics at the same supplementation protocol in all the tanks, may suggest some synergic requirement of a bacteria consortium to obtain a successful performance.

The LEfSe analysis showed some genera and species that can be considered as biomarkers for improved survival after AHPND infection in shrimp hatcheries. However, biomarkers found in this study cannot be considered as healthy biomarkers as we did not perform a comparison with non-infected controls. Only 6 out of 64 tanks were AHPND negative and survival of these 6 tanks was low (32.2 ± 18.4%), indicating that they were not suitable as a control for non-infected successful tanks. *Yangia pacifica*, *Mameliella alba*, and *Pelagibaca bermudensis*, together with other two ASVs (ASV-20 and ASV-80), corresponding to the genera *Roseobacter* and *Cognatishimia*, belonging to the *Rhodobacteraceae* family, were significantly enriched in the high-survival tanks. On the other hand, the genus *Gilvibacter* has been reported as a biomarker associated at early stages (zoea 2) in *P. vannamei* larvae (Wang H. et al., 2020). In this study, this genus was significantly enriched in the low-survival tanks. To the best of our knowledge, there are no reports of the association of *Gilvibacter* with AHPND or any disease affecting *P. vannamei*. Therefore, it could be recommended to study this genus more closely.

The following evidence sufficiently supported the biomarkers associated with high and low survival in shrimp hatchery tanks: (a) larvae samples used for the microbiota comparison were collected from the two groups of tanks with a marked and significant difference in survival at harvest despite both the groups were AHPND positive, (b) alpha- and beta-diversity indices were significantly different between the high- and low-survival tanks, especially at M3 stage, and (c) *Bacillota*/*Bacteroidota* ratios were different between the high- and low-survival tanks. The survival levels reported at both the groups of tanks could depend on the presence or absence of the consortium of biomarkers shown by the LEfSe analysis. It suggests that the biomarkers found in the high-survival tanks could improve the performance of the production cycles. However, due to the high prevalence of AHPND in most tanks, the biomarkers identified for low survival also deserve special attention. This study constitutes the first characterization of the microbiome of the *P. vannamei* larval stages affected by AHPND in a commercial hatchery. The results provide new insights into the microbiome-host relations and contribute to the development of effective probiotics to prevent significant losses in AHPND-affected larvae. Most probiotics in shrimp culture are identified through *in vitro* antagonism screening, which overlooks many bacterial strains. Therefore, this analysis contributes with knowledge directed to different approaches of probiotic discovery based on the *in vivo* identification of prospective probiotics that include modes of action other than just antagonism. This is based on detecting the composition of the natural bacterial consortium present at high survival and the identification of their synergic interactions requirement to obtain a successful performance.

## Data Availability Statement

The sequences from this study are available in the National Center for Biotechnology Information (NCBI) Sequence Read Archive (SRA) under accession number PRJNA800805.

## Author Contributions

GR and BB contributed with conceptualization, methodology, validation, formal analysis, data curation, visualization, writing, and original draft preparation of this study. GR contributed with software. BB contributed with supervision and project administration. GR, BB, RR, BA, FP, IB, LS, and LT contributed with investigation. BB, LS, and LT contributed with resources and funding acquisition. All authors contributed with writing, reviewing, and editing.

## Conflict of Interest

The authors declare that the research was conducted in the absence of any commercial or financial relationships that could be construed as a potential conflict of interest.

## Publisher’s Note

All claims expressed in this article are solely those of the authors and do not necessarily represent those of their affiliated organizations, or those of the publisher, the editors and the reviewers. Any product that may be evaluated in this article, or claim that may be made by its manufacturer, is not guaranteed or endorsed by the publisher.
