# Supplementary material for: Microbiome of Penaeus vannamei Larvae and Potential Biomarkers Associated With High and Low Survival in Shrimp Hatchery Tanks Affected by Acute Hepatopancreatic Necrosis Disease
Source: Front Microbiol. 2022 May 9;13:838640. doi: 10.3389/fmicb.2022.838640 (PMC9125206; doi:10.3389/fmicb.2022.838640)
Supplement: Supplementary file 1 [file Data_Sheet_1.docx]

**Supplementary Table 1.** Description of commercial feeds and probiotics supplemented during the production cycle.

| **Commercial feeds** | **Commercial probiotics** |
| --- | --- |
| Skretting PL | Epicin G2 |
| Frippak fresh | Sanolife mic |
| Zeigler Z Plus | Biozyn Aquapro |
| Advance Brine Shrimp | Sanolife Pro2 |

**Supplementary Table 2.** Description of samples of *P. vannamei* larvae infected with AHPND-causing bacteria selected for the microbiome comparison between high and low survival tanks, and summary of the sequencing information. AHPND-positive samples were assigned to two groups of survival conditions: high survival (samples collected from tanks with survival > 60%) and low survival (samples collected from tanks with survival < 60%).

| **Tank** | **Survival at harvest (%)** | **Sample** | **Larval stage** | **Survival condition** | **Number of ASVs** | **Number of clean reads** | **Goods coverage (%)** | **Error rate (%)** | **GC content (%)** | **Phred Q30 (%)** |
| --- | --- | --- | --- | --- | --- | --- | --- | --- | --- | --- |
| 1 | 83.5 | L5 | Postlarvae 4 | High survival | 401 | 142,272 | 99.998 | 0.03 | 53.76 | 88.45 |
|  |  | L6 | Postlarvae 7 | High survival | 372 | 162,977 | 99.997 | 0.03 | 52.61 | 88.36 |
|  |  | L7 | Postlarvae 10 | High survival | 360 | 150,414 | 99.998 | 0.03 | 53.19 | 88.60 |
| 2 | 71.2 | L8 | Mysis 3 | High survival | 409 | 168,544 | 99.996 | 0.04 | 52.25 | 85.16 |
|  |  | L9 | Postlarvae 4 | High survival | 457 | 157,480 | 99.993 | 0.03 | 54.13 | 88.82 |
|  |  | L10 | Postlarvae 7 | High survival | 373 | 166,231 | 99.998 | 0.03 | 53.83 | 88.68 |
|  |  | L11 | Postlarvae 10 | High survival | 388 | 134,468 | 99.992 | 0.03 | 53.57 | 88.73 |
| 3 | 61.6 | L12 | Mysis 3 | High survival | 358 | 161,410 | 99.997 | 0.03 | 53.69 | 88.79 |
|  |  | L14 | Postlarvae 7 | High survival | 380 | 165,966 | 99.999 | 0.03 | 54.17 | 88.90 |
| 4 | 61.4 | L16 | Mysis 3 | High survival | 415 | 160,241 | 99.997 | 0.03 | 53.82 | 88.72 |
|  |  | L17 | Postlarvae 4 | High survival | 379 | 143,608 | 99.996 | 0.03 | 53.60 | 88.77 |
|  |  | L18 | Postlarvae 7 | High survival | 340 | 133,503 | 99.996 | 0.03 | 53.22 | 88.14 |
|  |  | L19 | Postlarvae 10 | High survival | 289 | 136,840 | 100.000 | 0.04 | 53.19 | 85.46 |
| 5 | 68.43 | L20 | Mysis 3 | High survival | 382 | 163,255 | 99.997 | 0.03 | 53.98 | 88.37 |
|  |  | L21 | Postlarvae 4 | High survival | 454 | 161,390 | 99.998 | 0.04 | 53.48 | 85.41 |
|  |  | L22 | Postlarvae 7 | High survival | 377 | 145,647 | 99.994 | 0.03 | 53.74 | 88.51 |
|  |  | L23 | Postlarvae 10 | High survival | 451 | 168,784 | 99.997 | 0.03 | 53.50 | 88.14 |
| 6 | 68.1 | L26 | Postlarvae 4 | High survival | 376 | 135,568 | 99.998 | 0.03 | 53.50 | 88.48 |
|  |  | L27 | Postlarvae 7 | High survival | 335 | 144,642 | 99.999 | 0.03 | 53.32 | 87.70 |
|  |  | L28 | Postlarvae 10 | High survival | 395 | 157,809 | 99.999 | 0.03 | 53.77 | 88.34 |
| 7 | 60.2 | L32 | Postlarvae 4 | High survival | 356 | 158,595 | 100.000 | 0.03 | 53.82 | 88.23 |
|  |  | L33 | Postlarvae 7 | High survival | 434 | 157,924 | 99.997 | 0.03 | 53.68 | 88.47 |
| 8 | 25.0 | L38 | Postlarvae 4 | Low survival | 349 | 145,592 | 99.999 | 0.03 | 53.83 | 88.15 |
|  |  | L39 | Postlarvae 7 | Low survival | 358 | 160,587 | 99.999 | 0.03 | 52.39 | 88.48 |
|  |  | L40 | Postlarvae 10 | Low survival | 360 | 146,752 | 99.995 | 0.04 | 54.06 | 85.25 |
| 9 | 0.0 | L44 | Mysis 3 | Low survival | 309 | 130,040 | 99.996 | 0.03 | 53.87 | 88.31 |
|  |  | L45 | Postlarvae 4 | Low survival | 399 | 136,451 | 99.998 | 0.03 | 53.10 | 88.94 |
|  |  | L46 | Postlarvae 7 | Low survival | 397 | 147,445 | 99.996 | 0.03 | 54.41 | 88.79 |
| 10 | 0.0 | L47 | Mysis 3 | Low survival | 331 | 147,831 | 99.999 | 0.03 | 52.26 | 88.28 |
|  |  | L48 | Postlarvae 4 | Low survival | 445 | 151,410 | 99.998 | 0.03 | 54.04 | 89.05 |
|  |  | L49 | Postlarvae 7 | Low survival | 482 | 160,061 | 99.997 | 0.04 | 52.84 | 84.44 |
| 11 | 25.0 | L50 | Mysis 3 | Low survival | 347 | 141,911 | 99.999 | 0.03 | 53.27 | 88.46 |
|  |  | L51 | Postlarvae 4 | Low survival | 431 | 143,408 | 100.000 | 0.03 | 54.59 | 88.58 |
|  |  | L52 | Postlarvae 7 | Low survival | 435 | 153,395 | 99.998 | 0.04 | 52.90 | 84.50 |
|  |  | L53 | Postlarvae 10 | Low survival | 466 | 167,189 | 99.994 | 0.03 | 53.29 | 88.39 |
| 12 | 25.0 | L54 | Mysis 3 | Low survival | 370 | 134,186 | 99.999 | 0.03 | 53.72 | 88.46 |
|  |  | L55 | Postlarvae 4 | Low survival | 437 | 157,179 | 99.997 | 0.03 | 54.76 | 88.99 |
|  |  | L56 | Postlarvae 7 | Low survival | 452 | 139,258 | 99.996 | 0.03 | 53.79 | 88.71 |
|  |  | L57 | Postlarvae 10 | Low survival | 375 | 133,245 | 99.998 | 0.03 | 53.69 | 88.99 |
| 13 | 25.0 | L58 | Mysis 3 | Low survival | 376 | 140,622 | 99.999 | 0.03 | 54.14 | 88.83 |
|  |  | L59 | Postlarvae 4 | Low survival | 447 | 146,989 | 100.000 | 0.04 | 52.21 | 85.16 |
|  |  | L60 | Postlarvae 7 | Low survival | 381 | 130,641 | 99.998 | 0.04 | 52.03 | 85.08 |
|  |  | L61 | Postlarvae 10 | Low survival | 443 | 139,971 | 99.999 | 0.04 | 52.27 | 85.10 |
| 14 | 25.0 | L62 | Mysis 3 | Low survival | 342 | 138,007 | 99.995 | 0.03 | 53.57 | 88.94 |
|  |  | L63 | Postlarvae 4 | Low survival | 412 | 136,720 | 99.998 | 0.03 | 54.07 | 89.00 |
|  |  | L64 | Postlarvae 7 | Low survival | 407 | 134,369 | 99.999 | 0.03 | 53.88 | 88.83 |
|  |  | L65 | Postlarvae 10 | Low survival | 436 | 139,961 | 99.996 | 0.03 | 54.41 | 89.17 |

**Supplementary Table 3.** Relative abundance of top bacterial taxa of *P. vannamei* larvae at high and low survival tanks affected by AHPND.

| **Survival** | **Stage** | **Phylum** | | |  | **Family** | | | |  | **Genera** | | | | |  |
| --- | --- | --- | --- | --- | --- | --- | --- | --- | --- | --- | --- | --- | --- | --- | --- | --- |
|  |  | *Pseudomonadota* | *Bacteroidota* | *Bacillota* | **Percentage (%)** | *Rhodobacteraceae* | *Vibrionaceae* | *Flavobacteriaceae* | *Bacillaceae* | **Percentage (%)** | *Catenococcus* | *Vibrio* | *Bacillus* | *Marinibacterium* | *Gilvibacter* | **Percentage (%)** |
| High | M3 | 70 | 11 | 8 | 89 | 29 | 25 | 4 | 11 | 69 | 14 | 11 | 11 | 2 | 1 | 39 |
|  | PL4 | 73 | 12 | 6 | 91 | 33 | 12 | 8 | 5 | 58 | 4 | 8 | 5 | 10 | 0 | 27 |
|  | PL7 | 75 | 10 | 7 | 92 | 43 | 6 | 5 | 6 | 60 | 3 | 3 | 6 | 6 | 0 | 18 |
|  | PL10 | 82 | 5 | 4 | 91 | 22 | 41 | 2 | 3 | 68 | 24 | 17 | 3 | 4 | 0 | 48 |
| Low | M3 | 65 | 29 | 2 | 96 | 41 | 11 | 24 | 2 | 78 | 7 | 4 | 2 | 11 | 18 | 42 |
|  | PL4 | 68 | 23 | 3 | 94 | 40 | 8 | 14 | 3 | 64 | 5 | 3 | 3 | 18 | 8 | 37 |
|  | PL7 | 73 | 15 | 2 | 90 | 36 | 12 | 7 | 1 | 56 | 8 | 3 | 1 | 13 | 2 | 27 |
|  | PL10 | 80 | 10 | 2 | 92 | 38 | 23 | 4 | 1 | 66 | 11 | 11 | 1 | 7 | 1 | 31 |

**Supplementary Table 4.** Taxonomic assignment for ASVs identified as differentially abundant in the microbiome of *P. vannamei* larvae at high and low survival tanks affected by AHPND.

| **Survival** | **ASV level** | **Phylum** | **Class** | **Order** | **Family** | **Genus** | **Species** |
| --- | --- | --- | --- | --- | --- | --- | --- |
| High Survival Tanks | ASV_9 | *Bacillota* | *Bacilli* | *Bacillales* | *Bacillaceae* | *Bacillus* | *-* |
|  | ASV_35 | *Pseudomonadota* | *GammaPseudomonadota* | *Enterobacterales* | *Vibrionaceae* | *Vibrio* | *-* |
|  | ASV_8 | *Bacillota* | *Bacilli* | *Bacillales* | *Bacillaceae* | *Bacillus* | *-* |
|  | ASV_37 | *Pseudomonadota* | *AlphaPseudomonadota* | *Rhodobacterales* | *Rhodobacteraceae* | *Yangia* | *pacifica* |
|  | ASV_40 | *Cyanobacteria* | *Cyanobacteria* | *Chloroplast* | *-* | *-* | *-* |
|  | ASV_20 | *Pseudomonadota* | *AlphaPseudomonadota* | *Rhodobacterales* | *Rhodobacteraceae* | *Roseobacter* | *-* |
|  | ASV_18 | *Pseudomonadota* | *GammaPseudomonadota* | *-* | *-* | *-* | - |
|  | ASV_29 | *Bacillota* | *Bacilli* | *Bacillales* | *Bacillaceae* | *Bacillus* | - |
|  | ASV_42 | *Pseudomonadota* | *AlphaPseudomonadota* | *Rhodobacterales* | *Rhodobacteraceae* | *Mameliella* | *alba* |
|  | ASV_80 | *Pseudomonadota* | *AlphaPseudomonadota* | *Rhodobacterales* | *Rhodobacteraceae* | *Cognatishimia* | *-* |
|  | ASV_45 | *Pseudomonadota* | *AlphaPseudomonadota* | *Rhodobacterales* | *Rhodobacteraceae* | *Pelagibaca* | *bermudensis* |
|  | ASV_67 | *Pseudomonadota* | *AlphaPseudomonadota* | *Rhodobacterales* | *Rhodobacteraceae* | *Yangia* | *pacifica* |
|  | ASV_27 | *Patescibacteria* | *Gracilibacteria* | *JGI 0000069-P22* | *-* | *-* | *-* |
|  | ASV_73 | *Bacillota* | *Bacilli* | *Bacillales* | *Bacillaceae* | *Bacillus* | *-* |
|  | ASV_69 | *Pseudomonadota* | *AlphaPseudomonadota* | *Puniceispirillales* | *-* | *-* | *-* |
|  | ASV_84 | *Pseudomonadota* | *GammaPseudomonadota* | *Pseudomonadales* | *Halieaceae* | *Pseudohaliea* | *-* |
|  | ASV_58 | *Pseudomonadota* | *GammaPseudomonadota* | *Enterobacterales* | *Vibrionaceae* | *Vibrio* | *-* |
|  | ASV_39 | *Bacteroidota* | *Bacteroidia* | *Flavobacteriales* | *Flavobacteriaceae* | *Meridianimaribacter* | *flavus* |
|  | ASV_112 | *Bdellovibrionota* | *Bdellovibrionia* | *Bdellovibrionales* | *Bdellovibrionaceae* | *Bdellovibrio* | *-* |
|  | ASV_152 | *Pseudomonadota* | *GammaPseudomonadota* | *Enterobacterales* | *Shewanellaceae* | *Shewanella* | *-* |
|  | ASV_52 | *Bacillota* | *Bacilli* | *Bacillales* | *Bacillaceae* | *Bacillus* | *-* |
|  | ASV_169 | *Pseudomonadota* | *AlphaPseudomonadota* | *Rhodospirillales* | *-* | *-* | *-* |
|  | ASV_63 | *Pseudomonadota* | *GammaPseudomonadota* | *Pseudomonadales* | *Pseudohongiellaceae* | *Pseudohongiella* | - |
|  | ASV_164 | *Pseudomonadota* | *GammaPseudomonadota* | *Enterobacterales* | *Vibrionaceae* | *Vibrio* | *-* |
|  | ASV_128 | *Bacteroidota* | *Bacteroidia* | *Flavobacteriales* | *Flavobacteriaceae* | *Tenacibaculum* | *-* |
| Low Survival Tanks | ASV_7 | *Bacteroidota* | *Bacteroidia* | *Flavobacteriales* | *Flavobacteriaceae* | *Gilvibacter* | *-* |
|  | ASV_1 | *Pseudomonadota* | *AlphaPseudomonadota* | *Rhodobacterales* | *Rhodobacteraceae* | *Marinibacterium* | *-* |
|  | ASV_6 | *Bacteroidota* | *Bacteroidia* | *Flavobacteriales* | *Flavobacteriaceae* | *Spongiimonas* | *-* |
|  | ASV_16 | *Pseudomonadota* | *GammaPseudomonadota* | *Enterobacterales* | *Vibrionaceae* | *Catenococcus* | *-* |
|  | ASV_26 | *Bacteroidota* | *Bacteroidia* | *Chitinophagales* | *Saprospiraceae* | *-* | *-* |
|  | ASV_22 | *Pseudomonadota* | *AlphaPseudomonadota* | *Sneathiellales* | *Sneathiellaceae* | *Sneathiella* | *-* |
|  | ASV_59 | *Pseudomonadota* | *GammaPseudomonadota* | *Enterobacterales* | *Enterobacteriaceae* | *Escherichia-Shigella* | *-* |
|  | ASV_93 | *Pseudomonadota* | *GammaPseudomonadota* | *Pseudomonadales* | *Nitrincolaceae* | *Neptunomonas* | *-* |
|  | ASV_85 | *Pseudomonadota* | *AlphaPseudomonadota* | *Caulobacterales* | *Hyphomonadaceae* | *-* | *-* |
|  | ASV_65 | *Pseudomonadota* | *AlphaPseudomonadota* | *Rhizobiales* | *Methyloligellaceae* | *-* | *-* |

**Supplementary Table 5.** Results of the Bayesian t-test analysis performed for each biomarker obtained through the LEfSe analysis. Bayes Factor_10_ (BF_10_) indicated the relative performance in favor of H_1_ (different ASVs abundance between high and low survival tanks) over H_0_ (equal ASVs abundance between high and low survival tanks) provided by the data, and calculated as the ratio of the probability of H_1_ over the probability of H_0_ based on the data. BF_10_ higher than 1 showed evidence in favor of H_1_, with ranges of 1-3, 3-10, and >10 interpreted as weak, moderate, and strong evidence in favor of H_1._

| **Survival condition** | **ASV** | **Bayes Factor BF_10_** | **Error (%)** | **Evidence in favor of the H_1_ alternative hypothesis (different ASVs abundance between high and low survival tanks)** |
| --- | --- | --- | --- | --- |
| High survival tanks | ASV_45 | 272934796,6 | 3,15E-15 | Strong |
|  | ASV_29 | 1708950,6 | 1,29E-13 | Strong |
|  | ASV_9 | 841340,9 | 2,65E-13 | Strong |
|  | ASV_8 | 73552,2 | 7,81E-13 | Strong |
|  | ASV_112 | 21271,5 | 1,69E-12 | Strong |
|  | ASV_52 | 3116,2 | 7,75E-12 | Strong |
|  | ASV_80 | 1937,9 | 2,95E-11 | Strong |
|  | ASV_69 | 1612,8 | 4,91E-11 | Strong |
|  | ASV_73 | 741,2 | 2,63E-08 | Strong |
|  | ASV_42 | 384,7 | 6,80E-10 | Strong |
|  | ASV_37 | 109,5 | 4,93E-10 | Strong |
|  | ASV_84 | 51,9 | 1,62E-09 | Strong |
|  | ASV_67 | 49,2 | 1,85E-09 | Strong |
|  | ASV_164 | 36,7 | 3,40E-09 | Strong |
|  | ASV_35 | 29,2 | 4,79E-09 | Strong |
|  | ASV_27 | 19,1 | 7,29E-09 | Strong |
|  | ASV_128 | 14,1 | 8,46E-09 | Strong |
|  | ASV_39 | 11,6 | 8,68E-09 | Strong |
|  | ASV_18 | 7,2 | 6,26E-09 | Moderate |
|  | ASV_63 | 7,2 | 6,21E-09 | Moderate |
|  | ASV_40 | 6,6 | 5,37E-09 | Moderate |
|  | ASV_20 | 4,8 | 6,35E-09 | Moderate |
|  | ASV_169 | 4,0 | 1,19E-08 | Moderate |
|  | ASV_58 | 2,5 | 8,53E-05 | Weak |
|  | ASV_152 | 2,1 | 8,29E-05 | Weak |
| Low survival tanks | ASV_59 | 113938,0 | 6,34E-13 | Strong |
|  | ASV_93 | 157,5 | 8,00E-10 | Strong |
|  | ASV_22 | 49,2 | 1,86E-09 | Strong |
|  | ASV_1 | 29,4 | 4,41E-09 | Strong |
|  | ASV_6 | 24,9 | 5,77E-09 | Strong |
|  | ASV_16 | 19,9 | 7,08E-09 | Strong |
|  | ASV_7 | 11,3 | 8,66E-09 | Strong |
|  | ASV_85 | 10,6 | 8,56E-09 | Strong |
|  | ASV_65 | 8,9 | 7,89E-09 | Moderate |
|  | ASV_26 | 6,7 | 5,51E-09 | Moderate |


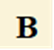

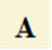

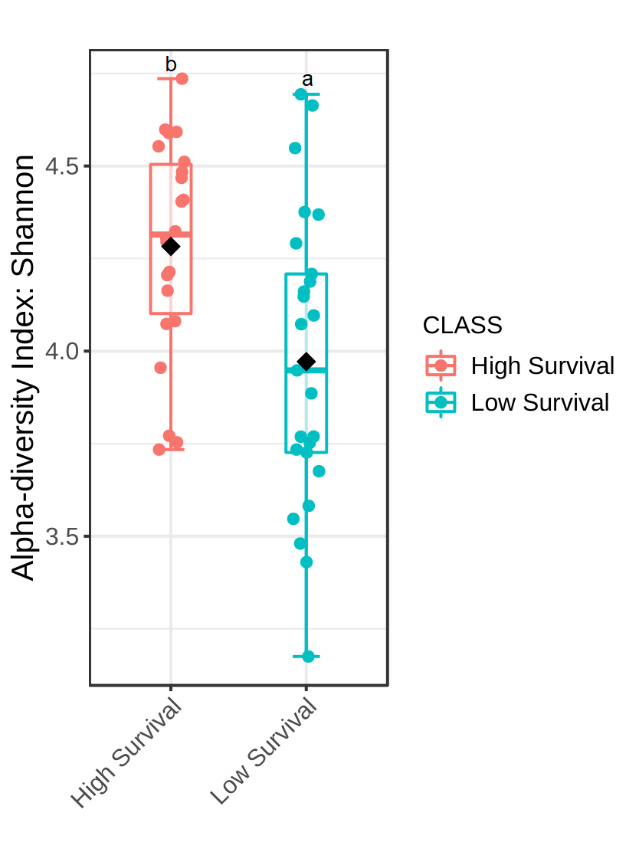

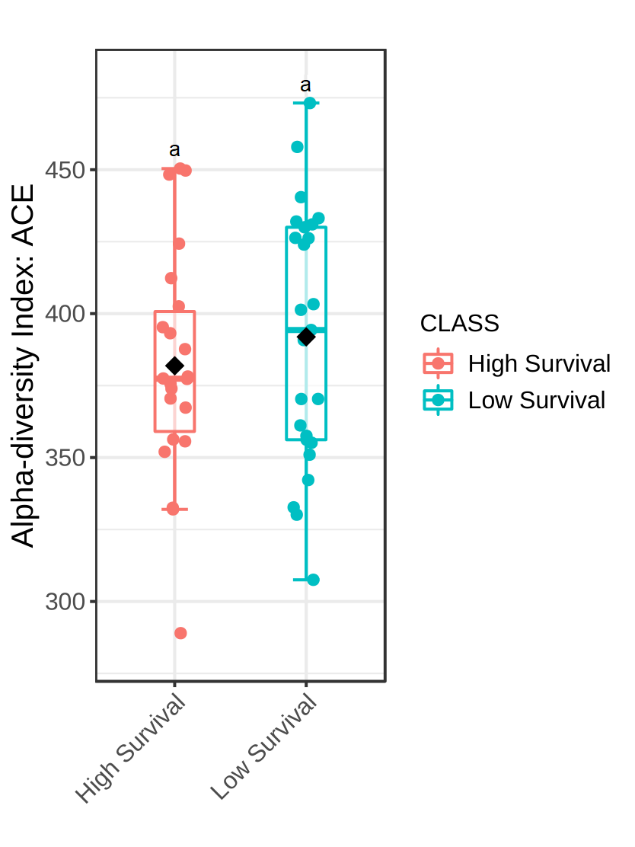


**Supplementary Figure 1.** Alpha diversity of the microbiome of *P. vannamei* larvae at high and low survival tanks affected by AHPND showing the global comparison between conditions (high and low survival tanks). (**A**) Shannon index. (**B**) ACE index. At each diversity index, pair of conditions with different letters are significantly different at p < 0.05, based on t-test.


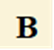

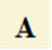

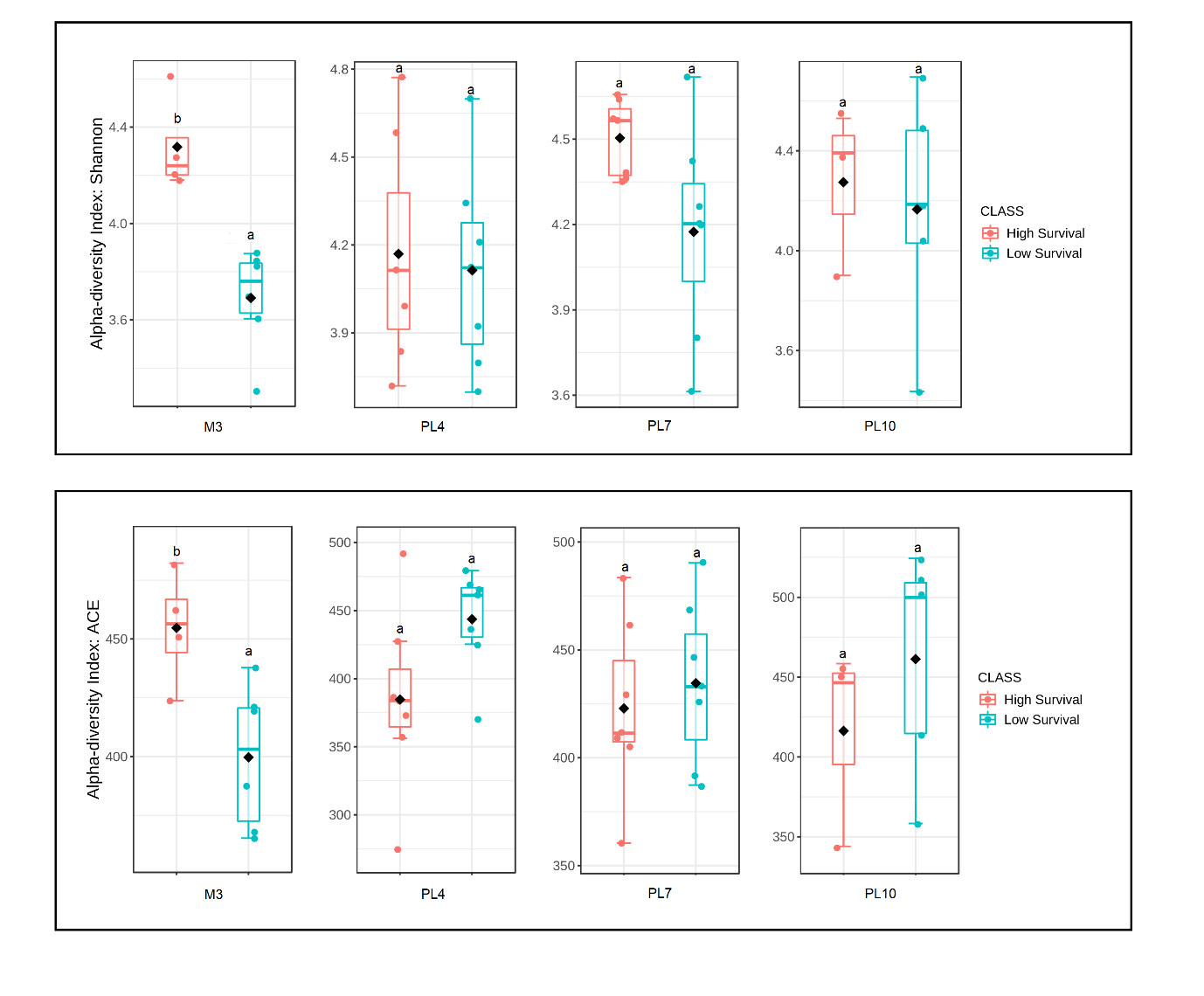


**Supplementary Figure 2.** Alpha diversity of the microbiome of *P. vannamei* larvae affected by AHPND through the larval stages at high and low survival tanks. (**A**) Shannon index individually compared at each stage between the high and low survival tanks. (**B**) ACE index individually compared at each stage between the high and low survival tanks. At each biological condition, pair of larval developmental stages with different letters are significantly different at p < 0.05, based on t-test.


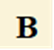

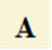

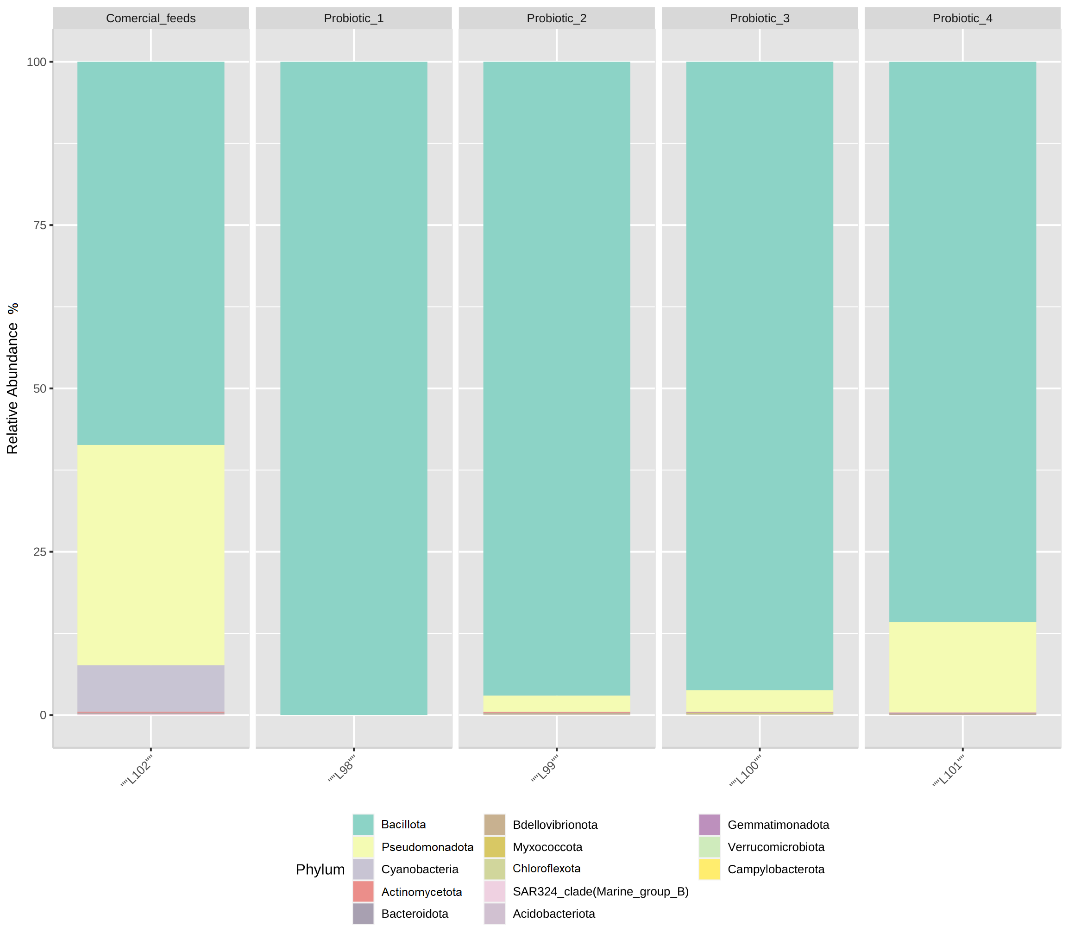

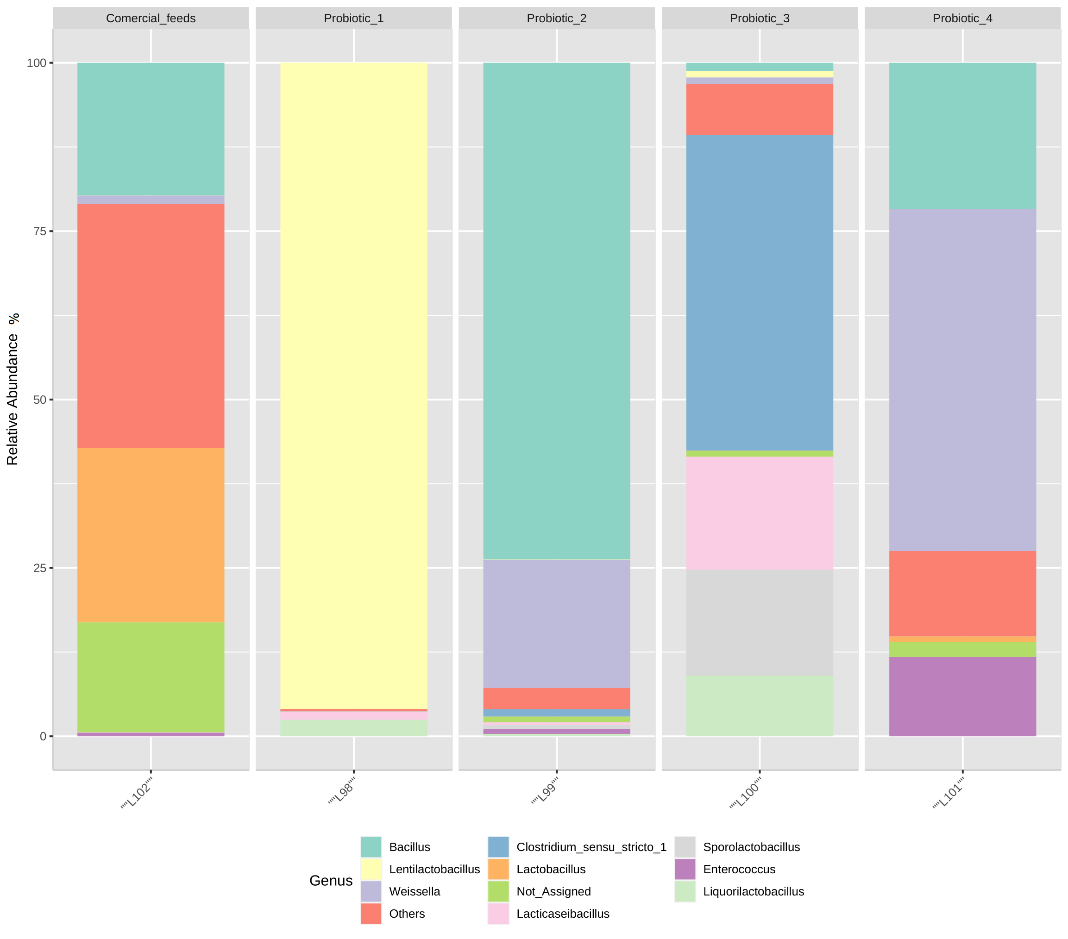


**Supplementary Figure 3.** Relative abundance (**A**) at phylum level and (**B**) genus level of the microbiome of commercial probiotics and commercial feeds.


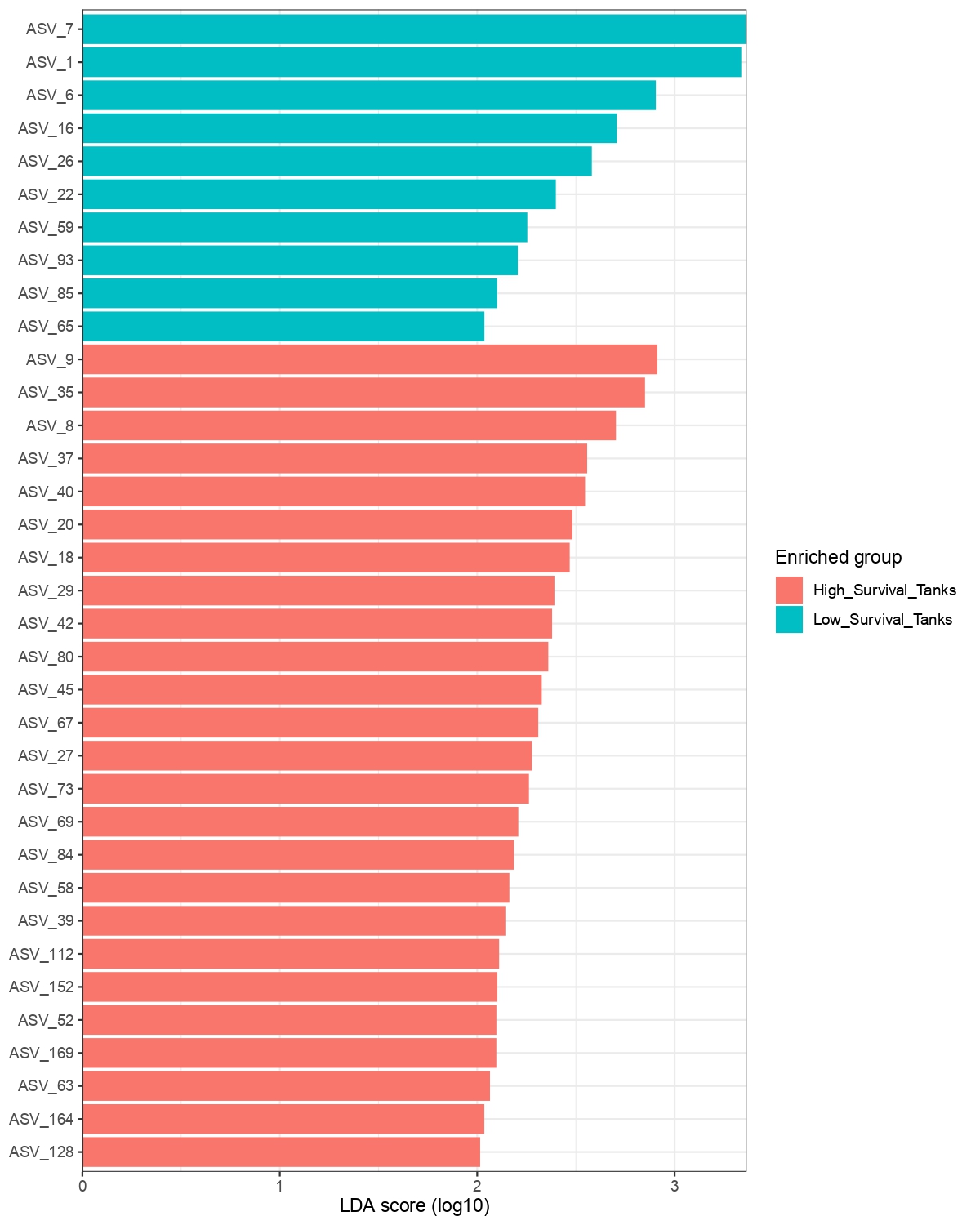


**Supplementary Figure 4.** Results of the differential abundance analysis using a linear discriminant analysis (LDA) effect size (LEfSe) of larvae collected from high and low survival tanks affected by AHPND**.** The length of the bar represents the effect size (LDA cutoff = 2) of all bacterial lineages at ASV level.
